# Low expression of TFPI-2 associated with poor survival outcome in patients with breast cancer

**DOI:** 10.1186/1471-2407-13-118

**Published:** 2013-03-15

**Authors:** Cheng Xu, Huijun Wang, Hongyu He, Fengyun Zheng, Yating Chen, Jin Zhang, Xiaoyan Lin, Duan Ma, Hongwei Zhang

**Affiliations:** 1 Department of Breast Surgery, Yangpu Hospital, Tongji University, Shanghai, 200090, China; 2 Children’s Hospital of Fudan University, Shanghai, 201102, China; 3 Department of General Surgery, Zhongshan Hospital, Fudan University, Shanghai, 200032, China; 4 Key Laboratory of Molecular Medicine, Ministry of Education, Department of Biochemistry and Molecular Biology, Institute of Medical Sciences, Shanghai Medical College, Fudan University, Shanghai, China

**Keywords:** Breast cancer, TFPI-2, Prognosis, Immunohistochemical staining, Survival analysis

## Abstract

**Background:**

The purpose of this study is to evaluate the prognostic value of TFPI-2 expression in breast cancer patients through examining the correlation between TFPI-2 expression and breast cancer clinicopathologic features.

**Methods:**

Immunohistochemical staining combined with digital image analysis was used to quantify the expression of TFPI-2 protein in breast tumor tissues. For evaluation of the prognostic value of TFPI-2 expression to each clinicopathologic factor, Kaplan-Meier method and COX’s Proportional Hazard Model were employed.

**Results:**

TFPI-2 expression was significantly correlated with tumor size, lymph node metastasis, histologic grade, clinical stage, and vessel invasion. More importantly, TFPI-2 expression was also associated with disease-free survival (DFS) of breast cancer patients. We found that patients with high TFPI-2 expression had longer DFS compared with those with low or negative expression of TFPI-2 (*P* <0.05, log-rank test). Cox’s regression analysis indicated that TFPI-2 expression, histologic grade, and vessel invasion might be significant prognostic factors for DFS, while TFPI-2 expression and histologic grade were the most significant independent predictors for tumor recurrence. Compared with the group with low/high TFPI-2 expression, the TFPI-2 negative group was more likely to have tumor relapse. The hazard ratio of DFS is 0.316 (*P* <0.01).

**Conclusions:**

Low or negative expression of TFPI-2 is associated with breast cancer progression, recurrence and poor survival outcome after breast cancer surgery. TFPI-2 expression in breast tumors is a potential prognostic tool for breast cancer patients.

## Background

Breast cancer remains the most frequent malignant tumor in women worldwide and is one of the leading causes of cancer-related mortality [1], while metastasis is the main reason for treatment failure of breast cancer. Better understanding of underlying mechanisms of breast cancer metastasis should contribute to the treatment and prevention. A common consensus is that breast cancer metastasis is a multi-gene involvement and multi-step process that lead to the reduction of intercellular adhesion and degradation of the extracellular matrix (ECM), a natural barrier against tumor metastasis [2,3]. Breast cancer cells secrete a variety of matrix metalloproteinases (MMPs) and active plasminogen, which hydrolyses ECM and facilitates tumor invasion and metastasis [4-6].

Human tissue factor pathway inhibitor-2 (TFPI-2) is a kunitz-type serine proteinase inhibitor, which is produced and secreted into ECM by endothelial cells, smooth muscle cells, fibroblasts, keratinocytes, and urothelium [7,8]. It is documented that TFPI-2 strongly prevents ECM hydrolysis by inhibiting plasmin and MMPs [9,10]. Recent studies show that the expression of TFPI-2 is down-regulated in several invasive tumor cell lines, including choriocarcinoma, glioma, prostate cancer, melanoma, fibrosarcoma, and pancreatic ductal adenocarcinoma, while exogenous expression of this gene in cancer cells can inhibit tumor growth and metastasis in vivo by modulating ECM remodeling and angiogenesis [11-16]. Recently, we show that TFPI-2 is down-regulated in highly invasive breast cancer cell lines due to hypermethylation of TFPI-2 promoter [17]. Similarly, several studies indicate that aberrant methylation of TFPI-2 gene promoter is found to be associated with a variety of malignant tumors [18-20]. These studies together suggest that TFPI-2 may be involved in tumor progression and have a potential prognostic value in cancer patients. The purpose of current study is to reveal the potential correlation between TFPI-2 expression level and clinicopathologic features by examining the expression level of TFPI-2 in tumor samples, in order to provide some meaningful insights to its value as a prognostic factor in breast cancer.

## Methods

### Specimen cohorts

Specimens were obtained from the female patients who were treated at department of breast surgery of Zhongshan Hospital affiliated to Fudan University from January 2005 to May 2008. From a total of 445 consecutive patients with operable primary breast cancer, we randomly selected 156 invasive breast cancer patients’ paraffin blocks of tumor tissues for our study (random numbers table) after excluding the patients with neoadjuvant chemotherapy or positive margins on histopathology. All patients received breast cancer surgery and standardized adjuvant therapy. Meanwhile, 40 benign breast tumor tissues were collected as controls. The selected breast cancer patients were divided into three groups according to cTNM staging system of American Joint Committee on Cancer (AJCC), including 40 patients in stage I, 74 patients in stage II, and 42 patients in stage III. In our specimens, 34 cases that had vessel invasion were defined as the presence of neoplastic emboli in two or more blocks [21].

We followed up the selected patients through phone or/and outpatient visits every month from one month after surgery till August 2011, and the follow-up was ended up with 118 patients with a median of 39 months (range of 2 to 75 months). 38 patients were lost in the follow-up. We found that 33 patients suffered from local recurrence or distant metastasis after surgery, in which local or regional recurrence was confirmed by histology and distant metastasis was detected by biopsy or imaging techniques. 7 patients died, with five of them due to breast cancer. 85 patients were found free of tumor recurrence. This study was approved by the Research and Ethical Committee of Zhongshan Hospital affiliated to Fudan University.

### Immunohistochemical staining

Immunohistochemical staining was performed by a two-step assay on 4-μm thick tumor sections. Briefly, tissue slides were de-paraffinized with xylene and re-hydrated through alcohol gradient washes. Antigen retrieval was carried out by immersing the slides in citric acid fix solution (pH 6.0) for 20 minutes at 95°C. The endogenous peroxidase activity was blocked by incubation in a 0.3% hydrogen peroxide buffer for 15 minutes. The sections were rinsed in Tris-HCl-buffered saline (pH7.6) and incubated with 3% bovine serum albumin to block nonspecific staining, and then incubated with a mouse polyclonal anti-TFPI-2 antibody (1:2000) overnight at 4°C in a humidified chamber. The mouse polyclonal anti-TFPI-2 antibody was generated by immunizing mice as previously reported [22]. The sections went through a stringent washing session before being incubated with the secondary antibody (horseradish peroxidase-conjugated rabbit anti mouse immunoglobin, EnVision Detection Kit A solution, Gene Tech, Hk) for 30 minutes at room temperature. Diaminobenzidine (EnVision Detection Kit B+C solution) was used as a chromogen, and sections were counterstained with hematoxylin. The placenta tissue sections were used as positive controls, and the buffer for dilution of primary antibody as a negative control. The staining intensity in cytoplasm was quantified as described below.

### Semi-quantitative analysis of immunohistochemical staining sections

All immunostained slides were analyzed by two pathologists independently in a blinded manner. Results from two pathologists were identical in most cases, and discrepancies were resolved by re-examination and consensus. No staining signal or staining signals present in less than 10% of tumor cells was considered as negative otherwise was considered positive. All the positive-staining sections were quantified using a computerized image system which was composed of OLYMPUS DP71 camera and OLYMPUS BX51 microscope, with the photo resolution of 13 million pixels. Six representative fields (200×objective) were picked up from hot-spot areas on every slide with identical settings (including do shading correction and white balance) for quantification of immunohistochemical densities.

**Figure 1:**
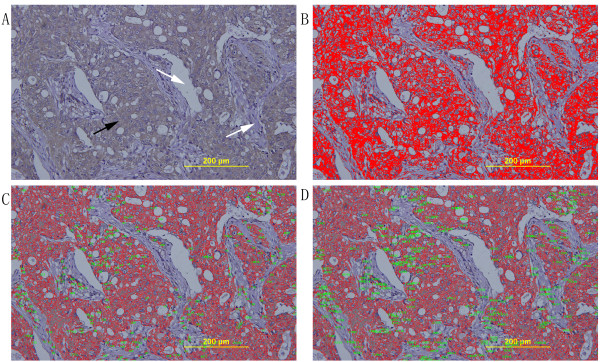
**Semi-quantification of immunostaining by digital image analysis. (A)** The original photograph acquired from tissue sections. Black arrow indicates the positive staining cells, and white arrow indicates the negative staining stroma area. (magnification×200). **(B)** Marking the sections. The positive staining cytoplasm that is chosen as the color of interest and masked with red color. **(C)** The area of positive staining which is marked by red lines (excluded the noisy spots less than 50 pixels). The exact number of positive staining area which labeled by green spots. **(D)** The area of positive staining which is marked by red lines (excluded the noisy spots less than 50 pixels). The exact number of positive staining IOD which is labeled by green spots. IOD divided number of positive staining area is mean-density that represented the concentration of TFPI-2 expression.

We used Image-Pro Plus 6.0 software (Media Cybernetics, Inc., Silver Spring, MD) for image analysis, which generated the integrated optical density (IOD) of TFPI-2 staining as well as the area of target protein distribution in each slide, as shown in Equation 1. In general, IOD value is proportional to the amount of target protein. The mean-density was obtained with IOD value divided by area (as shown in Equation 2), which represents the amount of target protein (TFPI-2) per unit area. Figure 1 shows representative images in Image-Pro Plus data analyses.

(1) $$\mathrm{IOD}=\int_{S}\mathrm{density}\left(x,y\right)\mathrm{ds}$$

(2) $$\mathrm{density}\left(\mathrm{mean}\right)=\mathrm{IOD}/\mathrm{area}$$

The level of TFPI-2 expression was represented by mean-density value which is obtained by averaging the density of six visions on each slide.

### Statistical analysis

The differences in clinicopathologic features between the TFPI-2 negative group and the positive group were determined using Pearson’s χ^2^ test. In the TFPI-2 positive group, the correlation between TFPI-2 expression and clinicopathologic characteristics was tested using T test or ANOVA. Disease-free survival (DFS) was defined as the time period from the date of surgery operation to the first recurrence (local or distant) or death from breast cancer without a recorded relapse. The survival curves of each group were estimated by Kaplan-Meier survival analyses, and the curves were compared using log-rank tests. In multivariate analysis, a COX’s Proportional Hazard Model was applied to determine whether a factor was an independent predictor of survival. All statistical tests were two-sided, and *P* values less than 0.05 were considered as statistically significant. The statistical analyses were performed using SPSS 18.0 software (SPSS Inc.).

## Results

### Immunohistochemical tissue staining

**Figure 2:**
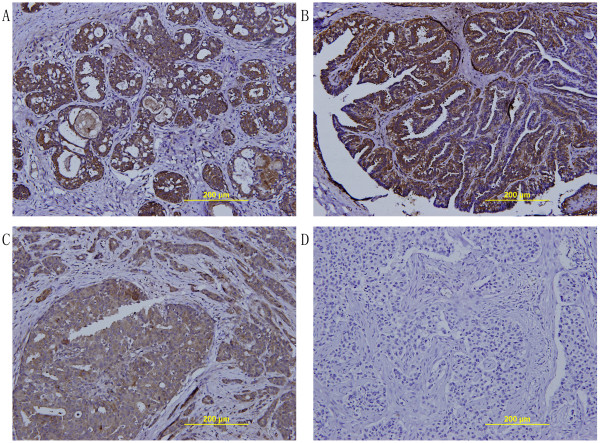
**Immunohistochemical staining for TFPI-2 expression in breast tumors. (A)** A representative TFPI-2 positive staining image using sections of hyperplasia of mammary glands. **(B)** A representative TFPI-2 positive staining image using sections of intraductal papillomas. **(C)** A representative TFPI-2 positive staining image using sections of breast cancer. **(D)** A representative TFPI-2 negative staining image using sections of breast cancer. (magnification×200).

We found that TFPI-2 staining was observed mainly on the cytoplasm of cells in breast glandular tissue or breast tumor tissue. Although sporadic positive staining was found on stroma areas, most of these areas showed negative staining. Strong positive staining could be observed in almost all benign breast tumors, while in breast cancer tissues, either positive staining or negative staining could be found. In general, the TFPI-2 staining tended to be weaker in breast cancer tissues than that in benign breast tissue, as shown in Figure 2 (A-D).

### Correlation of TFPI-2 Expression and clinicopathologic features

Altogether, we obtained 196 female patients in this study, including 156 breast cancer patients and 40 benign breast tumor patients. The median age of breast cancer patients was 54 years old (range of 29 to 95 years old). The median age of benign breast tumor patients was 38 years old (range of 21 to 55 years old). Almost all benign breast tumors exhibited high levels of TFPI-2 expression, with a mean of mean-density of TFPI-2 staining as 0.697 (95% CI 0.662-0.732). In the 156 breast cancer patients, 22.4% (35 of 156) of patients were TFPI-2-negative (including 19 cases with no staining signal and 16 cases with staining cells <10%), while the remaining 77.6% (121 of 156) showed variable levels of TFPI-2 expression, with a mean of mean-density of this cohort as 0.325 (95% CI 0.315-0.336).

We further stratified these 156 breast cancer patients as TFPI-2 positive and TFPI-2 negative groups according to the TFPI-2 staining of tumor sections. We found that, compared with patients with TFPI-2 positive breast cancer, patients with TFPI-2 negative had higher proportion of lymph node metastasis and poor differentiation in histology and more common vessel invasion. However, the differences of patient’s age, pathological type, clinical stage, and expression of hormone receptor and HER-2 in two groups did not appear to have any correlation with TFPI-2 expression (Table 1).

**Table 1:** Clinicopathologic features and expression of TFPI-2

	**TFPI-2 negative No.(%)**	**TFPI-2 positive N0.(%)**	**χ**^**2**^	**P value**
Age				
≤55	24(68.6%)	74(61.2%)	0.639	0.424
>55	11(31.4%)	47(38.8%)		
Tumor size				
≤2 cm	12(34.3%)	56(46.3%)	1.589	0.208
>2 cm	23(65.7%)	65(53.7%)		
Skin involvement^a^				
No	30(85.7%)	96(79.3%)	0.710	0.399
Yes	5(14.3%)	25(20.7%)		
LN metastasis				
No	9(26.5%)	70(58.3%)	10.766	0.001
Yes	25(73.5%)	50(41.7%)		
Unknown	1	1		
Histologic grade				
≤II	13(37.1%)	71(58.7%)	5.066	0.024
>II	22(62.9%)	50(41.3%)		
Vessel invasion				
Absence	12(35.3%)	109(90.0%)	46.528	0.001
Presence	22(64.7%)	12(10.0%)		
Unknown	1	0		
Clinical stage				
I	5(14.3%)	35(28.9%)	4.809	0.090
II	22(62.9%)	52(43.0%)		
III	8(22.9%)	34(28.1%)		
ER				
(-)	12(35.3%)	43(35.8%)	0.003	0.954
(+)	22(64.7%)	77(64.2%)		
Unknown	1	1		
PR				
(-)	15(44.1%)	63(52.5%)	0.745	0.388
(+)	19(55.9%)	57(47.5%)		
Unknown	1	1		
HER-2				
(-)	17(50.0%)	62(53.0%)	0.095	0.759
(+)	17(50.0%)	55(47.0%)		
Unknown	1	4		
Tumor type				
IDC^b^	29(82.9%)	98(81.0%)	0.062	0.803
Non-IDC^c^	6(17.1%)	23(19.0%)		

a skin involvement include: edema, redness, nodularity, or ulceration of the skin.

b IDC, invasive ductal carcinoma.

c Non-IDC include: invasive lobular carcinoma, mucinous or colloid carcinoma, medullary carcinoma, metaplastic carcinoma.

In the TFPI-2 positive breast cancer group, we compared the mean-density, which represent the level of TFPI-2 protein, with the clinicopathologic features including many common predictors of survival (Table 2). We found that multiple clinicopathologic features, such as tumor size, skin involvement, lymph node metastasis, histologic grade, clinical stage, and vessel invasion, were significantly correlated with the mean-density of TFPI-2 staining (Table 2, *P*<0.05). The result suggests that breast cancer patients with lower level of TFPI-2 tend to present more advanced cancer features such as larger tumor, skin involvement, positive lymph nodes, poorer histologic grade, later clinical stage, presence of vessel invasion and so forth. Nevertheless, patient’s age, pathological type, and expression of hormone receptor and HER-2 have little association with TFPI-2 protein levels. The results above are corresponding to the results in Table 1.

**Table 2:** Clinicopathologic features and level of TFPI-2 protein in the TFPI-2 positive group

	**Category**	**No. of cases**	**Meandensity of TFPI-2 Mean± SD**	**Statistics value**	**p value**
Age	≤55	74	0.324±0.061	t=0.302	0.764
	>55	47	0.327±0.052		
Tumor size	≤2 cm	56	0.351±0.061	t=4.948	0.001
	>2 cm	65	0.303±0.044		
Skin involvement^a^	No	96	0.336±0.057	t=4.252	0.001
	Yes	25	0.284±0.038		
LN metastasis	0	70	0.348±0.055	F=12.896^b^	0.001
	1-3	22	0.292±0.043		
	4-9	15	0.316±0.048		
	10-	13	0.272±0.038		
	Unknown	1			
Histologic grade	≤II	71	0.339±0.057	t=3.389	0.001
	>II	50	0.305±0.052		
Vessel invasion	Absence	109	0.333±0.054	t=5.172	0.001
	Presence	12	0.251±0.030		
Clinical stage^c^	I	35	0.375±0.053	Chi-Square=43.867^c^	0.001
	II	52	0.317±0.048		
	III	34	0.286±0.034		
ER	(-)	43	0.327±0.055	t=0.253	0.801
	(+)	77	0.324±0.059		
	Unknown	1			
PR	(-)	63	0.319±0.055	t=-1.211	0.228
	(+)	57	0.332±0.060		
	Unknown	1			
HER-2	(-)	62	0.333±0.059	t=1.361	0.176
	(+)	55	0.318±0.056		
	Unknown	4			
Tumor type	IDC^d^	98	0.324±0.057	t=0.488	0.626
	Non-IDC^e^	23	0.330±0.059		

a skin involvement include: edema, redness, nodularity, or ulceration of the skin.

b Post Hoc Multiple Comparisons(LSD) shows difference of TFPI-2 expression is only present in the group without LN metastasis and the other groups.

c Post Hoc Multiple Comparisons(Games-Howell) shows difference of TFPI-2 expression was significant among the groups (Nonparametric Test).

d IDC, invasive ductal carcinoma.

e Non-IDC include: invasive lobular carcinoma, mucinous or colloid carcinoma, medullary carcinoma, metaplastic carcinoma.

### TFPI-2 expression and survival

**Figure 3:**
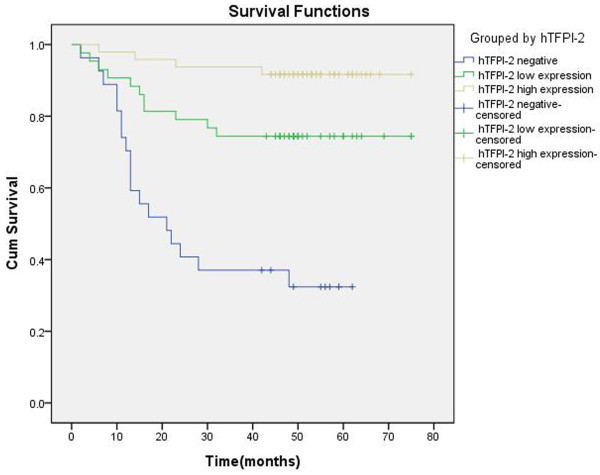
Kaplan–Meier analyses of the effect TFPI-2 expression on disease-free survival.

In a total of 118 patients with followed-up, we found that 91 cases with TFPI-2 positive breast cancer. We arbitrarily divided these 91 patients into two groups by median of TFPI-2 mean-density at 0.324. As shown in the Figure 3, patients with higher TFPI-2 expression (above the mean value) tended to have longer DFS compared with those with lower or negative expression. More importantly, we found that the group with negative TFPI-2 expression was statistically significantly associated with poorest DFS among these 118 patients (*P* < 0.05, log-rank test).

Furthermore, a multivariate COX ’s Proportional Hazard Model, in which tumor size, LN metastasis, histologic grade , vessel invasion, and TFPI-2 expression were included, showed that loss of TFPI-2 expression was an independent prognostic factor for DFS in breast cancer patients (Exp(B)=0.316, 95.0% CI 0.191~0.532, *P* <0.01). The results also showed that low histologic grade and vessel invasion were independent unfavorable factors for DFS, while poorer histologic grade appeared to have more significant impact on DFS (histologic grade ≤II vs. >II, Exp(B)=3.073, 95.0% CI 1.404~6.729, *P*=0.005) (Table 3).

**Table 3:** Multivariate Analyses of DFS (Backward Stepwise (Likelihood Ratio))

**Variable**	**HR**	**95%CI**	**P value**
Tumor size			
(≤2 cm vs >2 cm)	0.715	0.335 to 1.529	0.387
LN metastasis			
(No vs Yes)	1.028	0.459 to 2.298	0.947
Histologic grade			
(≤II vs >II )	3.073	1.404 to 6.729	0.005
Vascular invasion			
(No vs Yes)	0.534	0.219 to 1.298	0.166
TFPI-2			
(negative vs low/high expression)	0.316	0.191 to 0.523	0.001

## Discussion

Human tissue factor pathway inhibitor-2 (TFPI-2) is a kunitz-type serine proteinase inhibitor that plays a critical role in extracellular matrix (ECM) remodeling and homeostasis [9]. The ECM provides a scaffold for epithelial cells and contributes to cell apoptosis, proliferation, adhesion, migration, and differentiation, which are critical to tumor progression [2,23]. TFPI-2 inhibits the activity of plasmin and a variety of matrix metalloproteinases (MMPs), which are important to tumor invasion and metastasis. Our previous studies, along with the reports by others [17-19], indicate that dysregulation of TFPI-2 is associated with tumor progression.

Breast cancer originates in mammary epithelial cells, with a clear tendency to lymph node and blood metastasis. Before metastasis, cancer cells must degrade and destroy extracellular matrix and permeate the basement membrane [24]. It has been shown that breast cancer cells can secrete a variety of matrix metalloproteinases, and breast cancer cells with higher degree of malignancy appear to produce more types and amounts of MMPs [25]. Urokinase-type plasminogen activator (uPA) receptor, which lies on the surface of breast cancer cells, combines with free uPA in ECM and converts more plasminogen into plasmin [26-28]. Plasmin is also an activator of MMPs. Moreover, trypsin, chymotrypsin, plasma kallikrein can also activate pro-MMPs. TFPI-2 can inhibit activity of these enzyme, but also can directly inhibit the activity of MMPs [10,29]. The hydrolysis of ECM by plasmin and MMPs is the key steps for the tumor invasion and metastasis. In normal cells, TFPI-2 can inhibit plasmin and MMPs, reduce degradation of the ECM. In addition, TFPI-2 can inhibit vascular endothelial growth factor that is involved in promoting tumor angiogenesis by a negative feedback mechanism [30-32]. The reduction of TFPI-2 expression may weaken the inhibition of MMPs and plasmin, promote the development of malignant behavior in breast cancer. Early studies of our research group found that TFPI-2 showed low or negative expression in highly invasive breast cancer cell lines because the CpG islands in TFPI-2 promoter was hypermethylated, and DNA methylation in the promoter region induced inactive chromatin structure and decreased KLF6 binding to its DNA binding sequence [17]. Exogenous expression of TFPI-2 may inhibit the malignant behavior of breast cancer cell line MDA-MB-435 in nude mice [17]. These results suggest that TFPI-2 is inversely related to the ability of invasion and metastasis of breast cancer.

In our present study, we further investigated the correlation between TFPI-2 expression and clinicopathologic features of breast cancer. We found that breast cancer tissues tended to have weaker degree or less portion of TFPI-2-positive staining than benign breast tumor tissues. Compared with TFPI-2-positive breast cancer patients, the TFPI-2-negative group had higher proportion of lymph node metastasis and poor differentiation in histology and more common vessel invasion. The histopathological parameters were found to be significantly associated with TFPI-2 (*P*<0.05). These findings indicate that loss of TFPI-2 expression in breast cancer is likely to contribute to the permeation of cancer cells into the basement membrane and metastasis. In the TFPI-2-positive breast cancer patients, we found that lowered expression of TFPI-2 seemed to be associated with advanced progress of breast cancer like larger tumor size, skin involvement, positive lymph nodes, later clinical stage, presence of vessel invasion, poorer histologic grade etc. Further survival analysis indicates that patients with high TFPI-2 expression have longer DFS compared to the others with low or negative expression. Negative expression of TFPI-2 is significantly associated with poorest DFS in these 118 patients (*P* < 0.05, log-rank test).

The peak time for breast cancer recurrence and metastasis is 1~3 years after surgery [1]. Local recurrence and distant metastasis indicate the failure of treatment in breast cancer. It is believed that local recurrence rarely occurs independently, which is often a harbinger of distant metastasis. Although adjuvant therapies improved long-term survival in breast cancer patients, thousands of people died of metastasis. Thus, further study on breast cancer recurrence and metastasis is essential to breast cancer treatment. Traditionally, tumor size, LN metastasis, and histologic grade are still the most important prognostic indicators. However, we found some patients with a relatively early TNM stage suffered from local or distant metastasis in our follow-up process. Cox regression analysis was applied to determine significant prognostic factor. The result shows that TFPI-2 expression and histologic grade are the significant prognostic factors. Patients with lower TFPI-2 expression are more likely to relapse. Moreover, we found that the hazard ratio (Exp(B)) of DFS is 0.316 (*P* <0.01), indicating that the group with lower TFPI-2 expression may have about 3 times more risk of breast cancer relapse. The results suggest the patients with lower TFPI-2 expression should receive more effective systemic therapy to reduce tumor recurrence.

Tumor occurrence and development can be considered as the accumulation of gene mutations and epigenetic modifications. The predominant consequence of this accumulation is the activation of proto-oncogenes or silencing of tumor-suppressor genes [33]. Consistent with previous reports that TFPI-2 can inhibit the occurrence or development of malignant tumors through various mechanisms, our results show the expression of TFPI-2 in breast benign tissue is significant higher than that in breast malignant tumor, and the advanced extent of breast cancer is correlated with lower expression of TFPI-2. More importantly, we found that the patients with TFPI-2-negative are significantly associated with poorest DFS, and patients with higher TFPI-2 expression have better cumulative survival. These results together indicate that TFPI-2 may act as a tumor suppressor in the development of breast cancer and could well be considered as a novel biomarker for prognosis and therapy in breast cancer.

## Conclusions

Low or negative expression of TFPI-2 is associated with breast cancer progression, recurrence and poor survival outcome after breast cancer surgery. TFPI-2 expression in breast tumors is a potential prognostic tool for breast cancer patients.

## Competing interests

The authors declare that they have no competing interests.

## Authors’ contributions

CX, DM, HWZ conceived and designed the study. CX, HJW, FYZ and YTC performed the experiments. CX and HYH collected the clinical data. CX, HYH, JZ and XYL analyzed the data. CX, DM and HWZ wrote the paper. DM and HWZ supervised the study. All the authors read and approved the final manuscript.

## Pre-publication history

The pre-publication history for this paper can be accessed here:

http://www.biomedcentral.com/1471-2407/13/118/prepub
